# Public Health Implications and Risk Factors Assessment of *Mycobacterium bovis* Infections among Abattoir Personnel in Bauchi State, Nigeria

**DOI:** 10.1155/2015/718193

**Published:** 2015-01-31

**Authors:** A. S. Sa'idu, E. C. Okolocha, A. A. Dzikwi, A. A. Gamawa, S. Ibrahim, J. K. P. Kwaga, A. Usman, S. A. Maigari

**Affiliations:** ^1^Department of Veterinary Public Health and Preventive Medicine, Ahmadu Bello University, PMB 1013, Zaria, Kaduna State, Nigeria; ^2^Area Veterinary Clinic (Kofar Ran), Ministry of Animal Resources and Normadic Resettlement, Bauchi State, Nigeria; ^3^Department of Veterinary Medicine, Faculty of Veterinary Medicine, Ahmadu Bello University, PMB 1013, Zaria, Kaduna State, Nigeria; ^4^TB Laboratory, Department of Veterinary Medicine, Faculty of Veterinary Medicine, Ahmadu Bello University, PMB 1013, Zaria, Kaduna State, Nigeria; ^5^University of Maiduguri Teaching Hospital, PMB 1069, Maiduguri, Borno State, Nigeria

## Abstract

Bovine tuberculosis (bTB) is a chronic infectious and contagious zoonotic disease of domestic animals, wild animals, and humans. It poses a public health threat and economic losses due to abattoir condemnation of infected carcasses during meat inspection of slaughtered animals. Bovine tuberculosis is widespread in Africa including Nigeria affecting both cattle and humans, particularly Northern Nigeria. A prospective survey was conducted from June to August 2013 in the three Zonal abattoirs of Bauchi State, Nigeria. A total of 150 structured close-ended questionnaires were administered to abattoir personnel to assess their level of awareness of bTB. This study was aimed at determining the level of public health awareness, attitude, and practices of abattoir workers of bTB in Bauchi State, Nigeria. There was a statistically significant association between respondents' awareness of bTB and their occupational status, age, and duration of exposure to cattle carcasses (*P* < 0.05); the odds of being aware of bTB were 9.4, 7.3, and 2.1, respectively. In conclusion, these demonstrate the urgent need for public health authorities to intervene in bTB control. The risk of bTB transmission as indicated by the personnel's practices and awareness levels in Bauchi State could be prevented through the use of protective clothing (PPEs).

## 1. Introduction

Bovine tuberculosis (bTB) is a chronic infectious and contagious zoonotic disease of domestic animals, wild animals, and humans [1]. It also occurs in a wide range of mammalian species [2]. It is characterized by the formation of granulomas in tissues especially in the lungs, lymph nodes, liver, intestines, and kidney [3]. Tuberculosis is a major health problem, with 8-9 million new cases and 3 million deaths annually worldwide [4]. The majority of these occur in the developing nations. Because control and eradication programmes for animal tuberculosis are lacking in most African countries. In Nigeria, there have been limited studies to determine the prevalence and relationship between bovine and human TB especially with the emerging culture of eating improperly cooked beef and mutton, along with the drinking of unpasteurized fresh milk [5, 6]. Raufu and Ameh [7] reported an estimated annual economic loss from bTB in Nigeria and its environs (due to organ/carcass condemnation in cattle) of about 14-24 million Naira. So also [8] reported an economic loss of ₦13, 871,014 ($110,968) per annum with associated public health implications due to tuberculosis as major reasons for condemnations in some abattoirs in Western-Nigeria.

Bovine tuberculosis is caused by* Mycobacterium bovis* which is a member of* Mycobacterium tuberculosis* complex [9, 10]. The aetiological agents of mammalian tuberculosis, classified as members of the* Mycobacterium tuberculosis* complex (MTBC), include* Mycobacterium tuberculosis*,* M. bovis*,* M. microti*,* M. caprae*,* M. africanum*,* M. canettii*, and* M. pinnipedii*.* Mycobacterium africanum *consists of a rather heterogeneous group of strains isolated from humans in Africa [11].* Mycobacterium bovis*, otherwise known as the bovine tubercle bacillus, is the cause of bovine tuberculosis and the organism may be transmitted by aerosol or droplets of exudates containing the bacilli. It can be transmitted by ingestion of feed and water contaminated with urine, faecal material, or exudates from diseased animals that contain the tubercle bacilli [12]. In rare cases, humans can become infected with* M. bovis* via direct inoculation [13]. Referred to as Butcher's Wart (analogous to Prosector's Wart, which is caused by* M. tuberculosis* and is an occupational risk associated with performing autopsies), this skin lesion can occur in persons handling infected meat. It is very rare and generally self-limiting. Because* M. bovis *is either enzootic or found sporadically in much of the developing world, there is clearly a risk of cow to human transmission by either ingestion or inhalation [14]. As a result of the lack of surveillance data, the actual scope of the problem is unknown. However, from the public health perspective, eradication programs in cattle and universal pasteurization of milk remain the main stays of the prevention of a disease in humans that is caused by transmission from cows. These measures should be augmented by public education efforts explaining the dangers of consuming unpasteurized dairy products in areas where* M. bovis* disease in humans is more common.

The bovine tubercle bacilli is usually assigned to bTB in cattle and sometimes could be used to denote* M. bovis* of the tubercle bacillus irrespective of the host. Bovine tubercle bacillus has one of the broadest host ranges of all known pathogens. The species has been reported in domesticated and feral Bovidae. Other species in which the disease has been reported include goat, sheep, pig, horse, cat, dog, fennec fox, bison, buffalo, badger, wild and feral pig, antelope, camel, man, and nonhuman primates [15]. Cattle movements, particularly those from areas where bTB is reported, are the best predictors of disease occurrence [16].

## 2. Materials and Methods

### 2.1. Study Area

Bauchi State occupies a total land area of 49,119 km² representing about 5.3% of Nigeria's total land mass and is located between latitudes 9° 3′ and 12° 3′ north of the equator and longitudes 8° 50′ and 11° east of the Greenwich Meridian [17]. The state is bordered by Kano and Jigawa State to the north, Taraba State and Plateau State to the south, Gombe State and Yobe State to the east, and Kaduna to the west. The state is highly populated with cattle mainly owned by Fulani herdsmen. The cattle population is estimated at 1,789,000, about 13% of the Nigerian cattle population of 13,900,000 [18]. The state has a human population of 4,653, 066, which ranked 11th of the 36 states, density of 95 km^2^ (250 g/m^2^), and per capita income of $983 [19].

Bauchi State has a total of 55 tribal groups in which Hausa, Fulani, Gerawa, Sayawa, Jarawa, Bolewa, Karekare, Kanuri, Fa'awa, Butawa, Warjawa, Zulawa, and Badawa are the main tribes. This study was carried out in Bauchi, Katagum, and Misau Local Government Areas (out of the 20 LGAs), each representing the three senatorial zones as Bauchi south, Bauchi north, and Bauchi central with populations of 493,810, 295,970, and 263,487, respectively.

### 2.2. Questionnaire Distribution

A total of 113 structured close-ended questionnaires were retrieved from and analysed after the distribution to abattoir staff focused on assessing the level of knowledge, attitude, and practice of bTB transmission from slaughtered cattle to man and* vice versa*. Assessment of their level of awareness was based on biodata (demographic features), knowledge (zoonotic nature and symptoms of TB in humans), attitude (vaccination status of abattoir staff), practice (use of protection while handling carcasses), and other risk factors and scoring recall format of “0–8” was adopted as criteria for a respondent to be regarded for analysis as described by Waller et al. [20]. Personnel selection from each abattoir included in the study was also based on compliance with the interviewer and punctuality in daily abattoir activities.

### 2.3. Data Analysis

Data were analysed using statistical package for social sciences (SPSS) version 20.0. Chi- square (*χ* ^2^) was used to determine possible association between variables and* M. bovis*. Odds ratio (OR) and 95% confidence interval were calculated to measure the strengths of association between variables and bTB (*M. bovis*). Tables and bar charts were constructed using Microsoft Excel 2010. Values of *P* < 0.05 were considered significant.

## 3. Results

**Figure 1:**
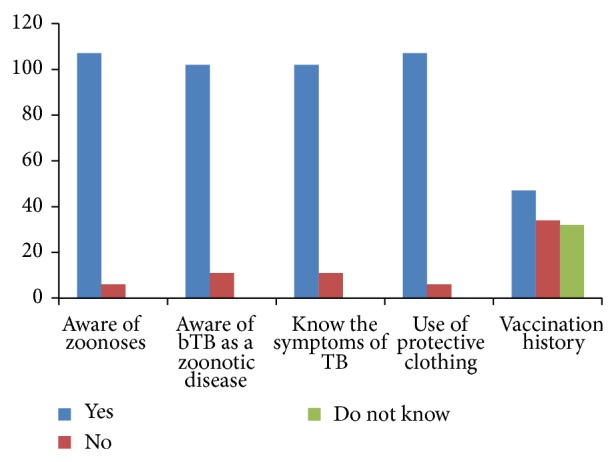
Level of respondents' awareness of bTB as a zoonotic disease, attitude, and practices of abattoir staff (by their knowledge of disease transmission from cattle, the zoonotic nature of bTB, common clinical signs of TB in humans, importance of use of protective clothing, and vaccination history, resp.), in Bauchi State, Nigeria.

Majority of the respondents had contact with cattle for over 3 years (81.40%) (Table 1). A significant number (57 (50.46%)) of them did not wear protective clothing when being in contact with cattle carcasses, despite the protection it gives against zoonotic transmission. This indicated their high risk of the zoonotic transmission, with the exception that a significant number (59.81%) of abattoir personnel consume boiled milk in recognition of the risk of contracting bTB and Brucellosis in fresh milk (unpasteurised milk). This study showed significant (*P* < 0.05) association between awareness of the respondents of bTB and their occupational status, age, and duration of exposure to cattle carcasses, and the odds of being aware of bTB by their level of awareness were 9.36, 7.29, and 2.06, respectively (Table 2). However, majority of the respondents believed in the importance of use of protective clothing while working, 94.7% (107/113), and 3.60% (4/113) had not. Likewise, on its zoonotic nature, 90.3% (102/113) knew that bTB can be contracted from cattle. Some of the respondents (41.6% (47/113)) had received childhood BCG vaccine while 30.1% (34/113) had not and 28.3% (32/113) had not known their vaccination status (Figure 1).

## 4. Discussion

In the public health risk analysis of bTB found in this study, there were more respondents in Bauchi abattoir (53.10%) compared to Misau and Katagum abattoirs. Butchers made up of most of the respondents were then followed by other abattoir staff and veterinarians. Many of them had not used protective clothing, despite the protection it gives against zoonotic transmission, clearly indicating their high risk of contracting bTB by occupation. This study showed significant (*P* < 0.05) association between awareness of the respondents (abattoir staff) of bTB and their occupational status, age, and duration of exposure to cattle carcasses. However, most of the abattoir staff believed in the importance of use of protective clothing while working, but very few of them did not know the importance. Likewise, on its zoonotic nature, most of them knew that bTB can be contracted from cattle. Some of the respondents had even received prophylactic BCG vaccine while others had not received it and few did not know their vaccination status. Therefore, this clearly indicated their awareness of bTB and its zoonotic risk, probably due to awareness campaign and researchers that visit the abattoir for sampling and data collection.

Public health (zoonotic) risk associated with the respondents' awareness and practices of bTB found in this study had agreed with the report of Cadmus and Adesokan [8] who reported an economic loss of N13, 871,014/annum with associated public health implications due to tuberculosis (7.95%) as major reasons for condemnations in some abattoirs in Western Nigeria. This also agreed with the report of Cadmus et al. [21] and Bello et al. [22], on management of slaughter houses in Northern Nigeria, who reported that none of the major abattoirs in Northern Nigeria met the minimum hygienic standard of operation as recommended by the Codex Alimentarius and they could not have supported the production of safe meat and meat products for human consumption.

The statistically significant association between awareness of the respondents of bTB and occupational status found in this study had agreed with the report of Tigre et al. [23] who carried out a survey on dairy farms in Ethiopia and reported a significant association (*P* = 0.001) between reactor cattle and human TB cases in households, indicating the high zoonotic risk of bTB among exposed individuals. Despite their awareness of the zoonotic risk, these figures had clearly indicated their high risk of contracting the disease due to exposure and negligence, as only few of the respondents (32.38%) used protective clothing in practical sense. Moreover, the exception was that a significant number (59.81%) of abattoir personnel of Bauchi State reported consuming boiled milk in recognition of the risk of contracting bTB and Brucellosis in fresh milk (unpasteurised milk). These findings were contrary to the report of Tigre et al. [23] in a cross-sectional study on public health implication of bTB in dairy cattle and dairy farm owners, in South Western Ethiopia, in which most of the respondents used either raw milk or nontreated soured milk, while some of the respondents consumed mixed (raw and cooked) meat and only few of the respondents were aware that cattle had TB among which only 25.7% of them recognised bTB as zoonotic. The differences between Ethiopians' and Nigerians' bTB awareness may be attributed to the differences in their norms and cultures as well as feeding habits.

*Mycobacterium bovis*, the cause of tuberculosis in cattle, sometimes causes disease in humans: the finding that majority of the respondents have had contact with cattle for over 3 years (81.40%) could be a risk factor that promotes transmission from cattle to humans. Transmission from cattle to humans is mainly by ingestion of raw cattle products from infected animals, and transmission by inhalation is possible when there is prolonged contact. This agreed with the report of Byarugaba et al. [24], a risk assessment study carried out in tuberculosis patients from Mbarara, major cattle keeping region in Uganda, to determine species of* Mycobacterium* responsible for the disease, whether* M. bovis *causes disease in humans. Conclusions from this study showed that large proportions of the respondents (90.27%) were aware of zoonotic bTB. However, some (32.38%) used protection while working with cattle carcasses. This showed their high risk of contracting bTB and other zoonoses endemic in the study area. These findings also demonstrated the urgent need for the state public health authorities to intervene. The risk of bTB transmission as indicated by the practices and awareness levels of the abattoir personnel in Bauchi State could be prevented through the use of protective clothing.

## Figures and Tables

**Table 1:** Demographic features and awareness of the respondents (abattoir staff, *n* = 113) of bTB in Bauchi State, Nigeria.

Variables	Number (%) of respondents
Abattoir location	
Hardawa (Misau)	20 (17.70)
Azare (Katagum)	33 (29.20)
Inkil (Bauchi State)	^*^60 (53.10)
Occupation of respondents	
Butchers	^*^45 (39.80)
Veterinarians	25 (22.50)
Animal scientists	12 (10.60)
Other abattoir staff	31 (27.40)
Age groups of respondents	
16–25	16 (14.29)
26–35	^*^44 (39.29)
36–45	31 (27.68)
≥46	21 (18.75)
Sex of respondents	
Males	^*^101 (90.99)
Females	10 (9.01)
Education level of respondents	
Primary	18 (16.07)
Secondary	21 (18.75)
Tertiary	^*^50 (44.64)
Informal	10 (8.93)
None	13 (11.61)

^*^Significant higher values.

**Table 2:** Risk analysis of some variables and other practices during work (use of protective clothing) among abattoir staff in Bauchi State, Nigeria.

Variables	Use of protective clothing by respondents		OR	95% CI on OR	*P* value
	Yes	No			
Occupation of respondents					
Butchers	12	33	0.34	0.13–0.90	
Veterinarians	20	2	9.36	1.86–47.17	
Animal scientists	6	5	1.13	0.28–4.47	^*^0.0001
Other staff	16	15	^**^1		
Age group of respondents					
16–25	9	7	7.29	1.51–35.23	
26–35	28	15	10.58	2.67–41.99	
36–45	14	16	4.96	1.20–20.55	^*^0.0028
≥46	3	17	^**^1		
Sex of respondents					
Males	47	51	0.26	0.05–1.33	0.161
Females	7	2	^**^1		
Duration of contact with cattle					
<1 year	8	3	3.2	0.80–12.87	0.1835
1–3 years	6	4	1.8	0.47–6.83	
>3 years	40	48	^**^1		
Duration of exposure to cattle carcasses					
<1 year	17	4	6.74	2.06–22.04	^*^0.0013
1–3 years	8	4	3.17	0.88–11.49	
>3 years	29	46	^**^1		
Awareness that the disease can be contracted from cattle					
Yes	52	51	2.03	0.36–11.62	0.4440
No	2	4	^**^1		
Awareness that humans contract TB from cattle					
Yes	52	46	4.52	0.91–22.39	0.0930
No	2	8	^**^1		

^*^ *P* < 0.05 regarded as significant; ^**^reference values (1).

## Recommendation

The government and relevant agencies should educate and enlighten the public on bTB preventive measures and enforce the use of protection (PPE) by all abattoir workers. Collaboration between professionals in the state should be encouraged to bridge the existing gap between the public health and bTB awareness.

## References

[1] Radostits O. M., Blood D., Hinchey K. (2007). *Eterinary Medicine: A Textbook of Diseases of Cattle Sheep, Pigs, Goats*.

[2] O'Reilly L. M., Daborn C. J. (1995). The epidemiology of *Mycobacterium bovis* infections in animals and man: a review. *Tubercle and Lung Disease*.

[3] Shitaye J. E., Tsegaye W., Pavlik I. (2007). Bovine tuberculosis infection in animal and human populations in Ethiopia: a review. *Veterinarni Medicina*.

[4] WHO (2002). World Health Organization report. *Fact Sheet*.

[5] Caffrey J. P. (1994). Status of bovine tuberculosis eradication programmes in Europe. *Veterinary Microbiology*.

[6] Shehu L. M. (1988). *Survey of tuberculosis and tubercle bacilli in Fulani herds, “Nono” and some herdsmen in Zaria area, Nigeria [M.S. thesis]*.

[7] Raufu I. A., Ameh J. A. (2010). Prevalence of bovine tuberculosis in Maidguri Nigeria—an abbattoire study. *Bulletin of Animal Health and Production in Africa*.

[8] Cadmus S. I. B., Adesokan H. K. (2009). Causes and implications of bovine organs/offal condemnations in some abattoirs in Western Nigeria. *Tropical Animal Health and Production*.

[9] Collins C. H., Grange J. M. (1983). The bovine tubercle bacillus. *Journal of Applied Bacteriology*.

[10] Pfeiffer U., Deviewa P. D. (2003). Tuberculosis in animals. *Clinical Tuberculosis*.

[11] Collins C. H., Grange J. M. (1987). Zoonotic implication of *Mycobacterium bovis* infection. *International Veterinary Journal*.

[12] Thoen C. O., Lobue P. A., Enarson D. A., Kaneene J. B., de Kantor I. N. (2009). Tuberculosis: a re-emerging disease in animals and humans. *Veterinaria Italiana*.

[13] Grange J. M. (2001). *Mycobacterium bovis* infection in human beings. *Tuberculosis*.

[14] Cosivi O., Grange J. M., Daborn C. J. (1998). Zoonotic tuberculosis due to *Mycobacterium bovis* in developing countries. *Emerging Infectious Diseases*.

[15] Francis J. (1958). *Tuberculosis in Animals and Man*.

[16] Gilbert M., Mitchell A., Bourn D., Mawdsley J., Clifton-Hadley R., Wint W. (2005). Cattle movements and bovine tuberculosis in Great Britain. *Nature*.

[17] Bauchi state Diary http://www.nigeriagalleria.com/Nigeria/States_Nigeria/Bauchi_State.html.

[18] FAO (2010). *Corporate Documentary Repository*.

[19] Census 2006, Nigeria. http://www.nigeriamasterweb.com/Nigeria06CensusFigs.html.

[20] Waller J., McCaffery K., Wardle J. (2004). Measuring cancer knowledge: comparing prompted and unprompted recall. *British Journal of Psychology*.

[21] Cadmus S. I. B., Adesokan H. K., Awosanya A. E. J. (2009). Public health issues and observations made during meat inspection at Bodija Municipal Abattoir, Ibadan, Oyo State, Nigeria. *Nigerian Veterinary Journal*.

[22] Bello M., Lawan M. K., Aluwong T., Sanusi M. (2015). Management of slaughter houses in Northern-Nigeria and the safety of meat produced for human consumption. *Food Control*.

[23] Tigre W., Alemayehu G., Abetu T., Deressa B. (2011). Preliminary study on public health implication of bovine tuberculosis in jimma town, South Western Ethiopia. *Global Veterinaria*.

[24] Byarugaba F., Grimaud P., Godreuil S., Etter E. (2010). Risk assessment in zoonotic tuberculosis in Mbarara, the main milk basin of Uganda. *Bulletin of Animal Health and Production in Africa*.

